# Biliary Viability Assessment and Treatment Options of Biliary Injury During Normothermic Liver Perfusion—A Systematic Review

**DOI:** 10.3389/ti.2022.10398

**Published:** 2022-05-30

**Authors:** Jule Dingfelder, Laurin Rauter, Gabriela A. Berlakovich, Dagmar Kollmann

**Affiliations:** Department of General Surgery, Division of Transplantation, Medical University of Vienna, Vienna, Austria

**Keywords:** normothermic machine perfusion, liver perfusion, biliary injury, biliary complication, biliary strictures, viability assessment, liver transplantation

## Abstract

In recent years, significant progress has been made in the field of liver machine perfusion. Many large transplant centers have implemented machine perfusion strategies in their clinical routine. Normothermic machine perfusion (NMP) is primarily used to determine the quality of extended criteria donor (ECD) organs and for logistical reasons. The vast majority of studies, which assessed the viability of perfused grafts, focused on hepatocellular injury. However, biliary complications are still a leading cause of post-transplant morbidity and the need for re-transplantation. To evaluate the extent of biliary injury during NMP, reliable criteria that consider cholangiocellular damage are needed. In this review, different approaches to assess damage to the biliary tree and the current literature on the possible effects of NMP on the biliary system and biliary injury have been summarized. Additionally, it provides an overview of novel biomarkers and therapeutic strategies that are currently being investigated. Although expectations of NMP to adequately assess biliary injury are high, scant literature is available. There are several biomarkers that can be measured in bile that have been associated with outcomes after transplantation, mainly including pH and electrolytes. However, proper validation of those and other novel markers and investigation of the pathophysiological effect of NMP on the biliary tree is still warranted.

## Introduction

**FIGURE 1:**
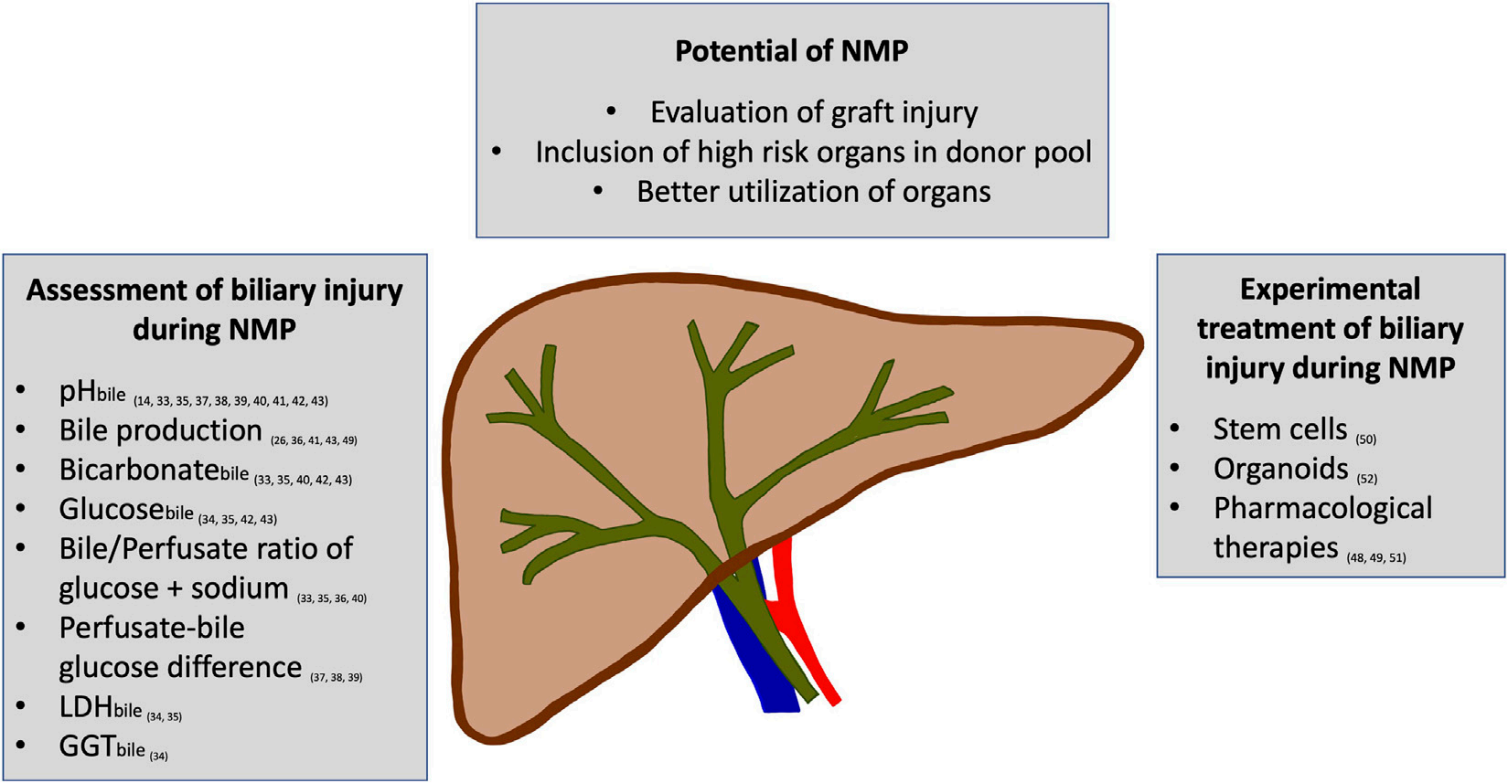
An overview of the potential of normothermic machine perfusion (NMP) to assess and improve biliary injury is displayed. Several parameters have been reported to allow the assessment of biliary injury during NMP. Additionally, the first experimental studies on treating biliary injury during NMP have shown promising effects.

Due to demographic change, there is a greater need for organs and the proportion of organs from older or unhealthier donors in the donor pool is growing. This leads to an aggravation of the already existing organ shortage and amplifies the need to use organs from so-called extended criteria donors (ECD). ECD include, for example, elderly donors, livers with steatosis, or donors with other comorbidities. Organs from a donation after circulatory death (DCD) donor are categorized as ECD since organs experience a harmful period of warm ischemia prior to explantation and enter the cold storage period already with an energy debt (1). A limiting factor of using ECD liver grafts is their susceptibility to postoperative complications, especially ischemic cholangiopathies (2), which are difficult to treat and are a leading cause for re-transplantation (3, 4). The pathophysiological processes involved in these ischemic type biliary lesions (ITBL) are complex and despite extensive research not completely understood. Factors that contribute to ITBL are ischemia and reperfusion, the associated inflammatory reaction, and the detrimental effect of non-physiologic bile composition in an already injured biliary system (4, 5). A certain degree of ischemic-reperfusion injury (IRI) is inevitable and can only be mitigated (6). Various approaches to diminish IRI in comparison to the standard preservation method of static cold storage (SCS) are currently in use. Many large transplant centers have implemented machine perfusion (e.g., hypothermic oxygenated perfusion (HOPE), normothermic regional perfusion (NRP), or normothermic machine perfusion (NMP)) to reduce organ injury. Machine perfusion aims to mitigate IRI by restoring the mitochondrial function prior to reperfusion or additionally ameliorating the injury by reperfusion of the organ in absence of immune cells (7–9). All machine perfusion strategies have shown a general benefit over SCS, however, they all have advantages and disadvantages depending on the indications they are used for. In several studies, HOPE and NRP have shown favorable effects on liver function after transplantation, including the development of ITBL (10, 11). A meta-analysis showed that HOPE was able to reduce the incidence of biliary strictures compared to SCS, while NMP was not (12). However, both HOPE and NRP are limited by their ability of organ assessment and treatment options. The implementation of NMP offers the chance for pharmacological treatment and viability assessment during perfusion (Figure 1) (13–16). The possibility of evaluating the biliary injury of a liver prior to transplantation or even treating it is thrilling. However, although NMP has found its place in the clinical routine, also because of its logistical benefits, literature on pathophysiological mechanisms and solid biomarkers to assess organ function are scarce. In this regard, the biliary tree is of high interest, as ITBL leads to increased morbidity and mortality of ECD organs. The benefit of HOPE or NRP is not based on assessment and there is currently no biliary-specific assessment marker that can be measured during HOPE. Therefore, the focus of this review is to summarize the available literature on the assessment and treatment options for biliary injury during NMP.

## Ischemia-Reperfusion and Biliary Injury

The diverse cells of the liver are all in different ways susceptible to one or all phases of IRI. The sinusoidal endothelial cells (SECs) in the liver for example are especially susceptible to cold ischemia (17). During reperfusion, the reintroduction of oxygen leads to an expression of danger-associated molecular patterns (DAMPs) and cytokines by SECs, and an imbalance of vasodilators and vasoconstrictors results in impaired microcirculation. DAMPs can activate Kupffer cells that secrete cytokines like tumor necrosis factor α leading to platelet adherence at SECs sending them into apoptosis (18). Cholangiocytes can handle periods of anoxia quite well compared to hepatocytes. However, they produce reactive oxygen species after reoxygenation and harbor fewer antioxidants like glutathione to compensate. Thus, bile duct cells are more susceptible to injury suffered through reoxygenation (17). Extensive damage to the bile duct epithelium can be found in almost every transplanted liver. In addition, the biliary regenerative capacity has been shown to be a crucial factor for long-term outcomes (5). The peribiliary glands (PBG) and peribiliary vascular plexus (PVP) play a critical role in the viability of the biliary system. Impaired blood supply through the PVP due to injured endothelium affects the regeneration after ischemia. The biliary progenitor cells that proliferate after damage to the biliary epithelium are mainly located in PBG deep in the bile duct walls. Whether or not the biliary system is capable to recover from IRI depends on the viability of the PBG and their blood supply through the PVP (5, 19, 20). The extra-hepatic biliary system and its connective tissue receive its blood supply only through the hepatic artery via microvascular networks. During ischemia, the endothelium is injured, therefore promoting thrombogenesis after graft reperfusion, thus further limiting blood supply (21).

The biliary tree is a complex and delicate system, damage to one part or one cell population often results in reactive changes or excessive proliferation of another. Therefore, biliary wound healing is a complex process (21). Deep wounds in the bile duct wall and consequently activation and transformation of myofibroblasts contribute to the formation of strictures. The bile itself contains growth factors and bile salts can induce several messenger pathways that either exacerbate damage or protect cholangiocytes. Bile composition and the effects of its different constituents play an important role in the pathophysiology of various cholangiopathies (21, 22) and is a potential target for therapeutic agents (23).

Ductular reaction (DR) is a form of intrahepatic wound repair mechanism (21). DR can be triggered by cholangiocyte injury in the smallest intrahepatic ducts or any change in the intrahepatic milieu, like increased pressure in the intra-biliary tract or via a strong stimulus of liver regeneration, e.g., after partial liver resection. DR is defined as hyperplasia of reactive bile duct tissue and is common in various biliary disorders. During DR transdifferentiation of various cells from the biliary tract has been described (21, 24).

## Impact of NMP on Biliary Injury

A summary of studies that consider biliary injury in the context of NMP can be seen in Table 1.

**TABLE 1:** Impact of NMP on biliary injury.

Author	MP	Design	Aim	Major findings
**Preclinical animal models**				
Op den Dries et al. (25)	Normothermic	Rat model	Impact of MP on bile duct preservation in DCD and non-DCD rat livers	GGT + LDH in bile were lower in NMP group; bicarbonate in bile and pH_bile_ higher in NMP group; ultrastructural changes most prominent in SCS-preserved DCD livers after reperfusion
		3 h preservation of 38 livers in 4 groups: non-DCD + NMP vs. non-DCD + SCS vs. DCD + NMP vs. DCD + SCS each followed by 2 h of *ex-situ* reperfusion		
		No report on systematic BD histology		
Westerkamp et al. (26)	Hypothermic	Rat model	Impact of machine perfusion on bile duct injury comparing different perfusion temperatures	Machine perfusion groups showed lower levels of transaminases + LDH; higher mitochondrial activity; better biliary function: bile production, bicarbonate secretion, pH_bile_; lower levels of biliary injury markers: GGT_bile_ + LDH_bile_; and less biliary epithelial injury in histological analysis
	Subnormothermic	30 DCD livers in 4 groups		
	Controlled oxygenated rewarming	6 h of SCS (Ctrl) plus either 1 h of HOPE, SNP, or COR; followed by 2 h of *ex-situ* reperfusion		
		Scoring system by op den Dries et al		
Boehnert et al. (27)	Normothermic acellular	Porcine model: 6 livers with 60 min WIT + 4 h SCS + 8 h NMP vs. 6 livers with 60 min WIT +12 h SCS vs. 60 min WIT + 4 h SCS; all with 12 h of whole blood reperfusion No report on systematic BD histology	Effects of NMP in porcine model of combined warm and cold ischemic injury with transplantation simulation	Reduced histologic biliary injury, reduced LDH in bile of the NMP group; higher bilirubin, phospholipids, and bile acids in bile of the NMP group
Liu et al. (28)	Normothermic	Porcine model10 h of NMP of 5 DCD livers (60 min WIT) vs. 5 SCS livers, 24 h reperfusion with whole bloodScoring systems by Hansen et al. + op den Dries et al	Impact of NMP on post-reperfusion outcomes in a transplant simulation model with DCD porcine livers	Biliary LDH and GGT higher in SCS; bicarbonate content in bile lower in SCS. Ki67 absent, and von Willebrand factor higher in SCS, indicating reduced biliary regeneration and increased platelet activation in SCS liver perivascular plexus
**Human trials**				
Mergental et al. (16)	Normothermic	Transplantation of 22 livers (12 DBD, 10 DCD) after NMP of 31 (17 DBD, 14 DCD) primarily discarded livers compared with control group (*n* = 44)	Feasibility of NMP as a method to push the boundaries to safe transplantation of highest risk organs	Similar graft and patient survival, higher incidence of EAD in NMP group, higher incidence of ITBL in NMP group (18% vs. 2%) but only the NMP group received routine magnetic resonance cholangiopancreatography imaging; Incidence of ITBL diagnosed by MRCP + clinical symptoms
		Median follow-up 542 days (456-641)No report on systematic BD histology		
		
**Randomized controlled trials**				
Nasralla et al. (30)	Normothermic vs. SCS	Human RCT	Effects of NMP in clinical practice compared to standard procedure (SCS)	NMP group showed
		121 NMP livers vs. 101 SCS		50% lower graft injury (transaminases, p< 0.001)
		Follow-up 12 monthsNo analysis of collected BD biopsies		50% lower organ discard rate (11.7% vs. 24.1%, *p* = 0.008), resulting in 20% increase in transplanted livers
				Reduction in bile duct complications statistically non-significant (11.1% in NMP DCD vs. 26.3% in SCS DCD on radiological imaging, *p* = 0.18)
				1 case of clinically relevant ITBL in each arm
Markmann et al. (31)	Normothermic vs. SCS	Human RCT	Effects of NMP in clinical practice compared to standard procedure (SCS)	Significant reduction of: EAD (18% vs. 31%, *p* = 0.01); histopathologic evidence of IRI after reperfusion (6% vs. 13%, *p* = 0.004); Incidence of ischemic biliary complications after 6 months (1.3% vs. 8.5%, *p* = 0.02) and 12 months (2.6% vs. 9.9%, *p* = 0.02); Higher use of initially accepted DCD livers (51% vs. 26%, *p* = 0.007)
		153 NMP livers vs. 147 SCS		
		Follow-up 12 months		
		No report on systematic BD histology		

BD, bile duct; COR, controlled oxygenated rewarming; DBD, donation after brain death; DCD, donation after circulatory death; EAD, early allograft dysfunction; GGT, γ-glutamyl transferase; HOPE, hypothermic oxygenated machine perfusion; IRI, ischemic reperfusion injury; ITBL, ischemic type biliary lesions; LDH, lactate dehydrogenase; NMP, normothermic machine perfusion; RCT, randomized control trial; SCS, static cold storage; SEC, sinusoidal endothelial cells; WIT, warm ischemic time.

In a rat model, op den Dries et al. compared NMP with SCS of DCD and non-DCD rat livers, followed by 2 h of reperfusion. They found increased bicarbonate and pH_bile_ and reduced GGT and LDH in the bile of the NMP group (25). Westerkamp et al. used a DCD rat model (*n* = 30) to compare hypothermic oxygenated perfusion (HOPE), sub-normothermic machine perfusion, and controlled oxygenated rewarming (COR) to SCS. All treatment groups showed an overall better outcome, lower levels of liver injury markers in perfusate, and better mitochondrial function. Furthermore, they showed higher bile production, bicarbonate secretion, and pH_bile_. Biliary injury was reduced, indicated by lower GGT and LDH in bile and by histological analysis (26).

In a porcine transplantation model that compared NMP livers to livers with long and short periods of SCS, Boehnert et al. showed reduced biliary injury, reduced LDH, and higher bilirubin, phospholipids, and bile acids in bile in the NMP group (27). In 2014, Liu et al. used a porcine model to investigate the impact of NMP on the biliary system. They described higher LDH and GGT in bile in the SCS group, lower bicarbonate in bile in the SCS group, and absent Ki67 and higher von Willebrand factor in immunofluorescence in the SCS group after reperfusion of the livers with whole blood. This indicates a positive effect of NMP on biliary injury and platelet activation, biliary regeneration, and bicarbonate secretion in porcine DCD livers (28). Mergental et al. described the NMP of 31 high-risk grafts that were deemed non-transplantable by two different surgeons (16, 29). A count of 22 livers were transplanted after viability assessment based on lactate clearance, perfusate pH, and the presence of bile production. A control group was matched in order to present the results within the framework of the centers’ contemporary outcomes. The control group did not receive high-risk grafts and comparisons were not powered to demonstrate any differences. Graft and patient survival were comparable, there was a higher incidence of early allograft dysfunction (EAD) (31.4% vs. 9.1%, *p* = 0.034) and ITBL in the NMP group (18% vs. 2%, *p* = 0.063). Only the NMP group received routine magnetic resonance cholangiopancreatography (MRCP) imaging. However, NMP was not able to prevent the development of ITBL in high-risk DCD grafts (16).

A variety of effects and benefits have been described above. These promising results all originate from non-randomized and sometimes even non-controlled trials, often with a small sample size. Recently, two larger-sized randomized controlled trials (RCT) have been published:

In 2018, Nasralla et al. published the first human RCT, comparing outcomes after NMP (*n* = 121) vs. SCS (*n* = 101). The NMP group showed 50% reduced graft injury measured by perfusate liver transaminases (*p* ≤ 0.001). Furthermore, a 50% lower organ discard rate (*p* = 0.008), resulting in a 20% increase of transplanted livers in the NMP group was observed. The reduction in MRCP diagnosed ITBL (11.1% in NMP-DCD and 26.3% in SCS-DCD grafts, *p* = 0.18) was statistically non-significant (30). The second human RCT was recently published by Markmann et al., they included 300 liver transplantations (randomized after initial acceptance—NMP *n* = 153, SCS *n* = 147). NMP grafts showed a reduction of EAD (18% vs. 31%; *p* = 0.01) and histopathologic evidence of IRI after reperfusion (6% and 13%; *p* = 0.004). NMP resulted in higher utilization of DCD livers initially accepted with 51% of transplanted grafts compared to 26% in the SCS group (*p* = 0.007). Despite the higher use of DCD organs in the NMP group, the incidence of ischemic biliary complications was reduced after 6 months (1.3% vs. 8.5%; *p* = 0.02) and 12 months (2.6% vs. 9.9%; *p* = 0.02). Ischemic biliary complications were defined as ITBL or bile leaks, which were confirmed either endoscopically or by magnetic resonance cholangiopancreatography. They did not mention if all patients or only symptomatic patients were examined (31).

In summary, several studies presented promising effects of NMP on LT in general, and partially regarding biliary complications. Additionally, NMP could increase the number of utilized organs. Nevertheless, our understanding of the mechanisms that influence biliary injury during NMP is incomplete. Many aspects of NMP are still vastly under-researched, such as the effect NMP has on cholangiocyte physiology. Existing preclinical studies investigated the effect either in animal models, which lack the equivalent of ITBL, or using discarded livers which represent a heterogeneous study group. Additionally, most clinical studies did not focus on mechanistic aspects and included ITBL development only as a secondary endpoint.

## Histological Scoring of Biliary Injury in Liver Transplantation

Possible surrogate endpoints for experimental studies are histological scoring systems. Histological tissue analysis reflects changes on a cellular level, which however cannot always be reliably translated into clinical outcomes.

Systematic histological workup of bile ducts most frequently refers to scoring systems published by Hansen et al. (32) or op den Dries et al. (5). The scoring of Hansen et al. assesses 7 features: mucosal loss, bleeding in bile duct wall, hyaline thrombi, vascular lesions, inflammation, arteriolonecrosis, and bile duct necrosis. The authors divided each feature into grades, depending on the severity of injury. The score has been developed by analyzing 93 transplanted livers of which 18 developed ITBL. Arteriolonecrosis, bile duct necrosis, vascular lesions, and intramural bleeding correlated with the development of ITBL, but arteriolonecrosis was the only parameter that was also associated with ITBL development in logistic regression analysis (32). Op den Dries et al. analyzed 128 bile duct biopsies obtained during liver transplantation. Injury severity scores were compared between grafts that later developed ITBL (16.4%) and grafts that did not. The score is a derivative of the Hansen score and assesses biliary epithelium, mural stroma necrosis, vascular injury, thrombosis, intramural bleeding, damage to periluminal and deep PBG, and inflammation. In the original publication of op den Dries et al., injury to the deep peribiliary glands and peribiliary vascular plexus was strongly associated with the development of ITBL. Contrarily, extensive loss of bile duct epithelium was observed in nearly every liver and was not indicative of ITBL development (5).

Both scores described above however have not yet been adjusted for well-known risk factors for the development of ITBL. Furthermore, it is not known to what extent a single feature of these scores contributes to the final risk of ITBL. Matton et al. selected the three histological parameters from op den Dries et al. that were predictive of ITBL development (stroma necrosis, injury to extramural PBG, and injury to PVP) to describe the bile duct injury (BDI) score. The score ranges between 0–7 and was developed in 23 human livers subjected to NMP but not transplanted, a cut-off of 4.75 was empirically defined using the median of the histological scores. The authors investigated their results prospectively in a subsequent clinical study during NMP of 6 livers of which 4 were transplanted (33). However, the level of evidence is currently not strong enough to recommend the universal application of this score for the prediction of ITBL.

The histological scoring systems discussed above can be considered useful tools if they are interpreted with the knowledge of their insufficient validation for the prediction of clinical outcomes in mind.

Furthermore, biomarkers that were identified with histological scoring systems as a surrogate endpoint for ITBL development should only translate into clinical decision-making after proper validation to prevent possibly transplantable livers from being discarded.

The accuracy of the described histologic scoring systems in predicting ITBL should be treated with caution but they offer at least a certain degree of objectivity and enable comparison of results.

## Biliary Assessment During NMP

Studies that focused on the assessment of biliary parameters during NMP are summarized in Table 2 and classified into animal studies, preclinical studies with discarded human livers, and clinical studies with subsequent transplantation after viability assessment.

**TABLE 2:** Biliary assessment during NMP.

Author	Design	Aim	Biliary viability criteria	Major findings
**Non-human studies**				
Linares-Cervantes et al. (35)	Porcine LT-model: transplantation after 4 h of NMP: 5 Non-DCD vs. 5 DCD30′ vs. 5 DCD70′ vs. 5 DCD120′No-PNF vs. 2 DCD120′PNF with 3-day follow-up	Investigation of biomarkers for graft function and preservation injury during NMP	Bile: LDH, pH, lactate, bicarbonate, glucose, sodium, b/p glucose + sodium ratio,	B/p sodium ratio ≥1.1 within 4 h of NMP strongly correlated with successful transplantation
			lactate + urea (hepatocellular)	
			No systematic BD histology	
Kesseli et al. (34)	Primate model: NMP of 4 DCD livers with 5 min WIT vs. 4 DCD livers with 45 min WIT	Characterization of trends in POC biomarker during NMP of primate DCD livers with short and long periods of WIT	Bile: LDH, glucose + sodium; Perfusate: FMN, GGT, lactate, ALT, ALP	Perfusate GGT might be predictive of livers that are at risk of developing cholangiopathies
	No follow-up		No BD biopsies collected	All WIT 45′ livers were nonviable and showed severe injury in the biopsies that progressed over time, GGT but not lactate discriminated between viable and nonviable livers
**Preclinical human studies**				
Eshmuminov et al. (36)	7-day NMP of 23 porcine livers with subsequent transplantation 3 h follow-up 7-day NMP of 12 human livers	Bile flow after stimulation as a viability criterion in long term NMP	B/p glucose ratio	8 human livers were viable after 7-day NMP; tazobac/methylprednisolone induce bile salt independent bile flow; UDCA is an adequate bile flow inductor; absence of bile flow despite stimulation is indicative of poor performance
			No systematic BD histology	Mean b/p glucose ratio in viable livers was <0.5 during all perfusions
**Human studies with transplanted livers**				
Watson et al. (37)	NMP of 47 livers (12 DBD, 35 DCD)		pH_bile_, biliary glucose,	Retrospect: Peak pH_bile_ < 7.5 identified three livers that later developed ITBL; peak pH_bile_ < 7.5 discriminated between livers with a high grade of circumferential stromal necrosis of septal bile ducts and livers without
	22 transplanted after evaluation Median follow-up 20 months (IQR: 8.4-24.7)		difference in glucose and pH in perfusate and bile (<10 mmol/L suggested relevant injury), proposed glucose challenge	
			Histology scoring by Hansen et al	
De Vries (14)	DHOPE-COR-NMP	Sequential hypothermic and normothermic perfusion, 3-months graft survival after viability testing, and transplantation of marginal livers that were primarily declined	pH_bile_	pH_bile_ > 7.45 after 150 min of perfusion used for the decision to transplant after NMP
	7 primarily declined DCD livers, 5 transplanted after viability testing Median follow-up 6.5 months (IQR: 5-10)		Bile duct biopsies were only obtained from the two discarded livers No systematic BD histology	
Van Leeuwen et al. (38)	DHOPE-COR-NMP of 16 DCD livers, 11 transplanted Median follow-up 12 months (IQR: 8-22)	Sequential hypothermic and normothermic perfusion as a tool to resuscitate and assess marginal grafts that were initially declined	pH_bile_ > 7.45 Histology scoring by Op den Dries et al	1 ITBL Difference between bile and perfusate pH, bicarbonate, and glucose are more predictive of bile duct viability than absolute values
Matton et al. (33)	6 h of NMP of 23 (18 DCD, 5 DBD) preclinical livers to identify cut-off values; 6 h NMP of 6 livers in a clinical trial to validate cut-off values, 4 transplanted after evaluation	Define the diagnostic accuracy of bile biochemistry for the assessment of BDI	pH_bile_ > 7.48 b/p glucose ratio <0.67 bicarbonate content in bile >18 mmol/L	Retrospect BDI score cut-off defined as 4.75 Biliary LDH <3689 U/l Bicarbonate in bile has highest PPV + NPV in discriminating between low and high BDI
	Median follow-up 8.3 months (IQR: 7.6-10.1)		Histology scoring adapted from op den Dries et al. (0-7)	
Ghinolfi et al. (42)	LT of older grafts (≥70 years) randomized	Role of NMP in graft and patient survival of recipients receiving grafts from octogenarian donors	pH_bile_, glucose, bicarbonate, sodium	NMP group showed reduced biliary injury in histological analysis; Not enough power for differences regarding graft- and patient survival between NMP and SCS
	10 NMP vs. 10 SCS		Histology scoring by op den Dries et al	
	Follow-up 6 months			
Cardini et al. (41)	NMP of ECD organs: 34 livers perfused; 9 livers discarded after evaluation during NMP	Introduce NMP into clinical practice, avoid nighttime transplantations, assessment of ECD livers	Bile production and pH_bile_ were assessed, but no cut-off values were specified	NMP feasible for clinical practice, logistic improvements compared to SCS, graft evaluation possible but not yet sufficient
	Mean follow-up 20 months (SD: ± 5.9)		No BD biopsies collected	No cases of ITBL
Weissenbacher et al. (43)	Transplantation after viability assessment of 45 livers out of 55 NMP	Value of biomarkers that are measured repeatedly as predictors for early graft function	Bile production was a mandatory criterion for DCD livers; Biliary parameters (pH, bicarbonate, glucose, and lactate) were only assessed during 15 perfusions	Bile parameters did not correlate with the occurrence of EAD or with liver function scores
	Follow-up 3 months		No BD biopsies collected	1 case of ITBL
Van Leeuwen et al. (40)	27 bile duct biopsies + bile samples of DCD livers during NMP	Influence of donor hepatectomy time on bile duct injury in histology, bile composition, and development of ITBL	Biliary bicarbonate, pH, and b/p glucose ratio	Donor hepatectomy time 50 min as cut-off showed 17% of high BDI with ≤50 min and 64% high BDI with ≥50 min hepatectomy time
	Retrospective analysis of 273 DCD transplantations with ITBL development within 2 years as an endpoint		Histology scoring	Livers with a shorter hepatectomy time and low BDI had more alkalotic bile and higher bicarbonate, b/p ratio of glucose did not differ significantly between livers with longer and shorter hepatectomy time
Gaurav et al. (39)	Bile samples of 100 livers (35 DCD, 65 DBD) after reperfusion, 12 cases of ITBL (5 clinically relevant) over a median follow-up period of 15 months (IQR: 11-20)	Retrospective analysis of bile samples after reperfusion	Blood-bile glucose difference, biliary sodium, pH_bile_	Blood-bile glucose difference of <6.5 mmol/L showed an 83% sensitivity and 62% specificity of predicting cholangiopathy
			Bile duct damage categorized into two groups (none to mild, moderate to severe) based on stromal necrosis	No correlation between bile chemistry and degree of bile duct damage
			ITBL was diagnosed by MRCP, in patients that showed increasing alkaline phosphatase or clinical symptoms	Sample numbers were underpowered to show subtle differences

ALP, alkaline phosphatase; ALT, alanine-aminotransferase; BD, bile duct; BDI, bile duct injury; b/p ratio, bile/perfusate ratio; COR, controlled oxygenated rewarming; DBD, donation after brainstem death; DCD, donation after circulatory death; DHOPE, dual hypothermic oxygenated reperfusion; ECD, extended criteria donor; FMN, flavin mononucleotide; GGT, γ-glutamyl transferase; ITBL, ischemic type biliary lesion; IQR, inter quartile range; LDH, lactate dehydrogenase; LT, liver transplantation; MRCP, Magnetic resonance cholangiopancreatography; NMP, normothermic machine perfusion; NPV, negative predictive value; PNF, primary non-function; POC, point of care; PPV, positive predictive value; WIT, warm ischemic time.

Two animal studies focused on predictive biliary markers during NMP for liver grafts that experienced different periods of warm and cold ischemia (34, 35). The bile/perfusate ratio (b/p ratio) of glucose and sodium (≤0.7 and ≥1.1, respectively) within 4 h of NMP was found to correlate with successful transplantation in a porcine model (35). In a non-human primate model (45′ warm ischemic time (WIT) vs. 5′ WIT) perfusate gamma-glutamyltransferase (GGT) levels discriminated between viable and nonviable livers with progressive injury. The authors concluded that GGT might be predictive of livers that are at risk of developing cholangiopathies (34). A long-term perfusion protocol for 7-day NMP was established by Eshmuminov et al. (36). The authors included 23 porcine livers of which 3 were transplanted. In a second phase 12 discarded human livers were evaluated and after 7 days of NMP 8 remained viable. The absence of bile flow despite stimulation with either tazobac, methylprednisolone, or UDCA was indicative of poor performance. B/p glucose ratio <0.7 was met by all porcine livers and the viable human livers. To use bile flow as a reliable viability criterion it should be complemented with bile composition parameters (36).

In preclinical human studies and clinical studies with subsequent transplantation that focused on biliary assessment and biliary complications the same markers frequently appeared in different constellations. Results were either validated prospectively with ITBL development or high grade of injury in bile duct biopsies as endpoints or in retrospective analysis. Biliary pH (pH_bile_) was one of the most used biliary markers. Proposed cut-off values were >7.48 (33), >7.5 (37), and >7.45 (14, 38). Rather than assessing absolute values several groups suggested assessing the values in bile in relation to perfusate values. Matton et al. proposed several cut-off values that were all determined during NMP of 23 preclinical livers and validated in a following clinical trial with 6 livers of which 4 were transplanted after viability assessment. Upon the determined values were a b/p glucose ratio <0.67 and LDH in bile <3689 U/L. Bicarbonate in bile of 18 mmol/L discriminated between low and high BDI [positive and negative predictive value (PPV and NPV) both >80%] (33). Van Leeuwen et al. made the observation that the bile pH, glucose, and bicarbonate of a liver that later developed ITBL were similar to the perfusate levels and proposed to use the difference between perfusate and bile as markers of biliary viability (38). Gaurav et al. assessed the bile composition of recipients after reperfusion and showed that a blood-bile glucose difference <6.5 mmol/L was predictive of ITBL development (83% sensitivity) (39). Watson et al. published a study that included NMP of 47 livers that resulted in 22 transplanted grafts after viability assessment. They discovered that differences in perfusate and bile glucose levels of <10 mmol/L indicated significant injury. A pH_bile_ <7.5 was identified retrospectively as a cut-off that discriminated between livers that later developed ITBL and livers that did not and in livers not transplanted the cut-off discriminated between high vs. low grade of circumferential stromal necrosis (37). Van Leeuwen et al. investigated the impact of hepatectomy time on bile composition and BDI in the histology of 27 DCD livers during NMP and validated their findings in a retrospective database analysis of 273 transplanted DCD livers with the development of ITBL within 2 years as the endpoint. Livers with longer hepatectomy time showed higher BDI, lower pH_bile_ and bicarbonate in bile (40).

Several studies measured bile composition during NMP but did not use it for assessment. Furthermore, some studies did not measure bile composition consistently making results difficult to interpret (41–43).

Although several biomarkers are already used for assessment, they can only point in a certain direction but do not enable reliable decision-making at this point. Most markers were defined in livers that were not accepted for transplantation, due to a variety of reasons. The defined cut-off values of these biomarkers have been applied in clinical trials with promising results, however, it is impossible to know at this moment if livers that did not meet the criteria would have indeed shown poor performance.

## Novel Biliary Biomarkers

Several promising experimental biomarkers assessing biliary injury have been described in the literature (Table 3). Currently, most of them are not established to be measured during perfusion. Novel biomarkers include microRNAs (miRNA) measured in different solutions as well as markers for tissue integrity and regeneration analyzed by immunofluorescence and immunohistochemistry. In 2013, Verhoeven et al. compared miRNA expression in graft preservation solution of 20 grafts that developed ITBL with 37 that did not. They found that the ratio of hepatocyte-derived (HD)miRNAs/cholangiocyte-derived (CD)miRNAs was higher in grafts that later developed ITBL (44). More recently Matton et al. investigated miRNA levels in perfusate and bile during NMP of 12 declined human liver grafts. The authors discovered that CDmiRNA-222 correlated with cholangiocellular injury and function reflected by LDH, bilirubin, and bicarbonate levels in bile. B/p glucose ratio correlated strongly with CDmiRNA-222 and HDmiRNA-122 in bile. Additionally, the ratio of HDmiRNA122/CDmiRNA222 at 30 min was predictive of injury of liver parenchyma after 6 h NMP (45).

**TABLE 3:** Novel Biliary biomarkers.

Author	Design	Aim	Biomarkers	Major findings
Verhoeven et al. (44)	Graft preservation solutions of 20 grafts that developed ITBL compared with 37 that did not	Assessment of miRNA composition and ratio at preservation is predictive of later ITBL development (defined as symptomatic and need of intervention, confirmed by cholangiopancreaticography)	CDmiRNA-30e	HDmiRNAs/CDmiRNAs significantly higher in grafts that developed ITBL
			CDmiRNA-222	
			CDmiRNA-296	
			HDmiRNA-122	
			HDmiRNA-148a	
			No report on systematic BD histology, ITBL assessed in liver wedge biopsies	
Matton et al. (45)	NMP (6 h) of 12 declined human liver grafts	Assessment of miRNAs in perfusate + bile of NMP liver grafts	CDmiRNA-222	CDmiRNA-222 in perfusate + bile correlated with cholangiocellular injury reflected by LDH in bile and cholangiocellular function reflected by bilirubin in bile
			HDmiRNA-122 and ratio	
			No report on systematic BD histology	
Liu et al. (46)	24 h of NMP of 10 discarded livers after 4–6 h of SCS	Characterization of lipid profile and assessment of graft function in steatotic discarded livers	Bile: volume, LDH, GGT, bicarbonate	Ki-67 staining increased in bile duct biopsies at the end of NMP indicating cholangiocyte and PBG regeneration
			Ki-67	
			Scoring systems by Hansen et al. + op den Dries et al	
De Jong et al. (47)	*Ex vivo* model of bile duct biopsies from discarded donor livers	PBG role in recovery of bile ducts post-ischemia	HIF1-α	Stem cells out of PBG can proliferate and transform to mature cholangiocytes after biliary injury
			VEGF	
			Glut-1	
			Ki-67 (proliferation)	
			CK19 (cholangiocytes)	
			Sox9 (endoderm progenitor)	
			Nanog (undifferentiated Stem cells)	
			CFTR (mature cholangiocytes)	
Franchitto et al. (20)	Retrospective analysis of 62 bile duct biopsies from transplanted patients compared to 10 control ducts	Investigation of PBG phenotype, integrity of PVP, and expression of VEGF-A by PBG	VEGF-A	PBG in transplanted ducts contain more progenitor cells, express more VEGF-A and VEGF-R2
			VEGF-R2	
			HIF	
			Histological scoring system by Hansen et al. and op den Dries et al	

BD, bile duct; CD, cholangiocyte derived; GGT, γ-glutamyl transferase; HD, hepatocyte derived; HIF, hypoxia inducible factor; ITBL, ischemic type biliary lesions; LDH, lactate dehydrogenase; miRNA, microRNA, NMP, normothermic machine perfusion; PBG, peribiliary glands; PVP, perivascular plexus; VEGF, vascular endothelial growth factor.

In 2018, Liu et al. investigated liver function and regeneration during 24 h of NMP in 10 discarded livers. The authors described regeneration of cholangiocytes and PBG during NMP of steatotic livers indicated by increased Ki-67 staining in BD biopsies (46). In an *ex vivo* bile duct model, De Jong et al. investigated the regenerative reaction of stem cells from PBG to biliary injury. PBG started to proliferate and transform within the first 24 h after reoxygenation which caused an increase in cholangiocytes and forming of epithelial monolayers. As a reaction to hypoxia, hypoxia-inducible factor-1α expression was increased followed by activation of metabolic and pro-angiogenic pathways characterized by expression of vascular endothelial growth factor (VEGF) and Glut-1 (47). In 2019, Franchitto et al. published a retrospective analysis of 62 bile duct biopsies from transplanted patients and compared them to 10 donor bile duct biopsies that did not experience ischemia. The authors described more progenitor cells in PBG of transplanted ducts, also VEGF-A and VEGF-R2 expression were increased (20).

## Therapeutic Approaches During NMP to Improve Biliary Injury

An overview of therapeutic approaches targeting biliary injury can be seen in Table 4.

**TABLE 4:** Therapeutic approaches during NMP to improve biliary injury.

Author	Design	Aim	Major findings
Goldaracena et al. (48)	Porcine transplantation model: 4 h of SNMP of 5 livers with anti-inflammatory and endothelial-protective agents vs. 4 h of NMP of 5 livers vs. 6 h of SCS	Improvement of NMP by applying strategies to reduce the activation of proinflammatory cascades	Serum ALP and total bilirubin levels were lower, with significantly lower bilirubin
	3-day follow-up		
	No systematic histological scoring		
Boteon et al. (49)	6 h of NMP with vs. without a combination of drugs that enhance lipid metabolism 5 livers per group	Efficacy of lipid metabolism enhancement during NMP on defatting and improvement of functional recovery	Treatment group: Higher bile production and higher pH_bile_ in defatted livers
			Down-regulation of oxidative stress markers, immune cell activation, and release of inflammatory cytokines
	No BD biopsies collected		Reduction in tissue triglycerides (38%) and macro-vesicular steatosis (40%)
Tian et al. (50)	DCD rat livers with WIT = 30min received BMMSCs, HO-1/BMMSCs, or neither during 4 h of NMP; Transplant model	Investigate the repair effect of HO-1/BMMSCs applied during NMP on biliary injury in a DCD rat transplantation model; Investigation of the underlying mechanisms	In the HO-1 group liver function and bile duct histology was improved; cell apoptosis was reduced; defective epithelium was restored through a large number of regenerative cells; Repair effect was inhibited through inhibition of Wnt signaling
	7 days postoperative follow-up		
	No systematic histological scoring		
Haque et al. (51)	12 h NMP of discarded DCD livers: 3 with tPA in HA at t = 0.5 h compared with 7 controls; 2 split grafts with 1 lobe tPA and 1 lobe control	Reconditioning of discarded DCD livers with tPA during NMP	Lower PVP and mural stroma injury score (0.67 and 1.3 vs. 2.0 and 2.7) using the Hansen et al. and op den Dries et al. histological scoring systems
Sampaziotis et al. (52)	Cholangiocyte organoids applied in mouse model and during human NMP	Investigate the feasibility of human cholangiocyte organoids for regenerative therapy during NMP	Cholangiocytes of human organoids can adapt to cellular environment, extrahepatic bile duct derived cells were able to repair intrahepatic bile duct injury
	No BD biopsies collected		Organoid-injected livers produced bile with higher pH and volume

ALP, alkaline phosphatase; ALT, alanine-aminotransferase; AST, aspartate-aminotransferase; BD, bile duct; BMMSC, bone marrow mesenchymal stem cell; DCD, donation after circulatory death, GGT, γ-glutamyl transferase; HA, hepatic artery; HO-1, heme oxygenase-1; NMP, normothermic machine perfusion; PVP, perivascular plexus; SNMP, subnormothermic machine perfusion; tPA, tissue plasminogen activator; WIT, warm ischemic time.

NMP offers the unique opportunity to treat livers under near physiologic conditions outside the human body. Several experimental studies have been published. In a porcine transplantation model, Goldaracena et al. described lower alkaline phosphatase and bilirubin levels during sub-normothermic machine perfusion with different anti-inflammatory agents, among others with a protective effect on endothelial cells (48). Boteon et al. described a higher volume of bile and higher pH_bile_ in livers treated with lipid metabolism enhancing pharmacological agents during NMP compared to standard NMP. Furthermore, liver grafts in the treatment group showed reduced activation of immune cells and release of inflammatory cytokines (49). Additionally, oxidative stress markers, macrovesicular steatosis, and tissue triglycerides were reduced. Tian et al. discovered that administration of Heme Oxygenase-1-modified bone marrow mesenchymal stem cells (HO-1/BMMSCs) during NMP of rat livers lead to improved liver function, bile duct histology, restored epithelium, and reduced cell apoptosis (50). Haque et al. investigated the reconditioning of discarded DCD livers with tissue plasminogen activator administration during NMP. The authors described lower PVP and mural stroma injury scores after treatment with a tissue plasminogen activator (51). In 2021, Sampaziotis et al. made the exciting discovery, that cholangiocyte organoids can be used to repair damage in the biliary tree independent from their region of origin. Livers that were injected with organoids produced more bile with a higher pH_bile_ (52).

## Summary

Machine perfusion of liver grafts received tremendous attention in recent years, leading to a number of publications with promising outlooks. Ultimately, the research objective is a safe increase in the number of transplantable organs. To meet this goal, mitigation of IRI, therapeutic graft improvement, and graft assessment have emerged as the main machine perfusion-based approaches.

Most well-established viability assessment protocols mainly focus on hepatocellular criteria. However, biliary complications are one of the main challenges in liver transplantation and biliary viability criteria are lagging behind hepatocellular criteria. While the development of this field is very promising, one weakness has to be addressed. Without standardization of protocols, definitions, and sample collection heterogeneous data will be reported and results will be difficult to interpret. Especially regarding biliary complications, results from previous studies should be further validated in prospective studies, with clear primary endpoints and appropriate follow-up periods.

A variety of preclinical and clinical studies introduced different biomarkers which can be used to assess the injury and regenerative capacity of the biliary system. The majority of parameters were analyzed in small pilot studies that differ greatly in their study design. Markers of interest in preclinical studies were either determined in comparison to historical cohorts or correlated with surrogate parameters in form of histological grading systems and injury markers. The screening for ITBL and its definition varied widely in clinical studies with some performing routine cholangiopancreatography (MRCP or ERCP) alone and others in combination with clinical symptoms and/or cholestatic laboratory parameters. As for sample collection, a comprehensive description of methods helps to put results into context. In some cases, pH_bile_ and bicarbonate were not reported due to contact with ambient air. Several groups suggested to cover secreted bile with mineral oil to achieve better comparability (14, 33). Nevertheless, rather than excluding results obtained with different protocols, they should be reported, and methods thoroughly described.

Currently available evidence on biliary injury from experimental and clinical studies looks very promising. First clinical machine perfusion trials reported increased graft utilization, with comparable clinical outcomes. Just recently, the benefit of NMP regarding biliary complications was highlighted in a large randomized control trial (31), further emphasized by a higher rate of transplanted DCD grafts.

However, machine perfusion has its limits and cannot yet undo extensive damage to the organ that has already occurred (e.g., after long ischemic times, etc.). Notably, it does offer the opportunity for therapeutic interventions. Only future studies will determine if therapeutic options such as organoids, mesenchymal stem cells, and novel targeted therapeutic agents can be used to further increase organ utilization.

In conclusion, normothermic machine perfusion is a thrilling opportunity to treat the organ. Each step towards the extension of the donor pool needs to be accompanied by careful graft assessment to ensure patient safety. Every additional organ available for transplantation is a gain and with further improvement in already promising biliary viability assessment, liver transplantation in the future, without its Achilles’ heel, seems within reach.
